# Supplementary material for: Patterns of Intron Gain and Loss in Fungi
Source: PLoS Biol. 2004 Nov 30;2(12):e422. doi: 10.1371/journal.pbio.0020422 (PMC532390; doi:10.1371/journal.pbio.0020422)
Supplement: Table S1 — Also available at http://genes.mit.edu/NielsenEtAl/. (4.3 MB ZIP). [file pbio.0020422.st001.zip › NielsenEtAl/html/512.html]

AN8283.1.NCU02624.1.MG04432.1.FG01000.1


```
 CLUSTAL W (1.82) Multiple Sequence Alignments - Introns Inserted


Sequence 1: NCU02624.1	555 aa
Sequence 2: MG04432.1	526 aa
Sequence 3: FG01000.1	526 aa
Sequence 4: AN8283.1	526 aa
Alignment Length: 560 aa
Number Identitical Residues: 261 aa
Alignment Score (without introns) 13618


MG04432.1 	MGLLQDTTG-PLVDAFYQLGTGAQVGVAFVSFIFLSVFFHVAQQIFFKNPHEPPVVFSWF
NCU02624.1	MGILQVVAG-PLSQQFSQLGTVSQIGVAIASFLFVAVVLNVLQQFLFKKPNEPPLVFHWF
FG01000.1 	MGLLQELAGHPLAQQFQELPLGQQVGIGFAVFLVLSVVLNVLNQLLFRNPNEPPMVFHWF
AN8283.1  	MGLVSLVLD-NVCERCSALSVWALSGLGLLSVIIIAVVLNVLRQILFKNPNEPPVVFHWF
          	**::.   .  : :    *      *:.:  .:.::*.::* .*::*::*:***:** **

MG04432.1 	PVVGSTVTYGKDPPQFFRD------------------------MAKK0YGNIFTFILLGK
NCU02624.1	PLIGSTITYGMDPPRFFKENREKVNPPTASCWATRELGLLTISALEQ~YGDCFTFILLGK
FG01000.1 	PFVGSTITYGMDPPTFFRE------------------------NRAK0HGDVFTFILLGK
AN8283.1  	PFIGSTISYGIDPYKFFFN------------------------CRAQ0YGDIFTFVLLGK
          	*.:***::** **  ** :                           : :*: ***:****

MG04432.1 	KTTVYIGTEGNEFILNGKLRDVNAEEIYGPMTTPVFGKDVVYDCPNAKLMEQKK~FMKIA
NCU02624.1	KTTVYVGPKGNDFILNGKIRDVNAEEIYTVLTTPVFGKDVVYDCPNSKLMEQKK0FMKIA
FG01000.1 	KTTVAVGPAGNDFILNGKLKDVCAEEIYTVLTTPVFGKDVVYDCPNAKLMEQKK0FMKIA
AN8283.1  	KTTVYLGTKGNDFILNGKLKDVCAEEVYSPLTTPVFGRHVVYDCPNAKLMEQKK0FVKYG
          	**** :*. **:******::** ***:*  :******:.*******:******* *:* .

MG04432.1 	LTTEAFRSYVPIIADEVSSYLKRTPAFKGPSGVVNIPPKMAEITIFTASHALQGKEIRDQ
NCU02624.1	LTTEAFRQYVPIISDEVTSYLKRTADFKGKSGIVDIPPKMAQITIFTASHALQGKEIRDK
FG01000.1 	LTTEAFRSYVPIISSEVRDYFKRSPDFKGKSGIADIPKKMAEITIFTASHALQGSAIRSK
AN8283.1  	LTSDALRSYVQLITAEVEDFAQKSSVFQNAKGVFDVSRTIAEITIYTASRSLQGKEVRDK
          	**::*:*.** :*: ** .: :::. *:. .*: ::. .:*:***:***::***. :*.:

MG04432.1 	FDETLADLYHDLDMGFHPVNFKLHWLPLPRNIRRDKAQKTIAKIYMDTIQRRRAKGKDSE
NCU02624.1	FDETLADLYHDLDMGFSPINFMLHWAPLPWNNRRDYAQRTVAKIYMDTIKERRARG-ETG
FG01000.1 	FDESLAALYHDLDMGFTPINFMLHWAPLPWNRKRDHAQRTVAKIYMDTIKERRAKGNNES
AN8283.1  	FDSTFAELYHDLDMGFAPINFMLPYAPLPHNRKRDAAQRKMAETYMEIIKERRKSGEKKD
          	**.::* ********* *:** * : *** * :** **:.:*: **: *:.**  *..  

MG04432.1 	AKDMMYHLMNSTYKNGTPVPDHEIAHMMIALLMAGQHSSSSTSSWIMLRLASRPDIMEEL
NCU02624.1	AQDIMWHLMNSTYKGDVPVPDHEIAHMMIALLMAGQHSSSSTSSWIMLRLASRPDIMEDL
FG01000.1 	EHDMMKHLMNSTYKNGIRVPDHEVAHMMIALLMAGQHSSSSTSSWIMLRLAQYPHIMEEL
AN8283.1  	SEDMVWNLMSCVYKNGTPLSDEEIAHMMIALLMAGQHSSSSTLSWILLHLARHPEIVEEL
          	 .*:: :**...**..  :.*.*:****************** ***:*:**  *.*:*:*

MG04432.1 	YQEQVRALGADLPPLRYEDLANLPLHLAVIKETLRLHAPINSILRAVKQDLPVPGTNYVI
NCU02624.1	YNEQVKNLGADLPPLTYEDLAKLPLHAAIVKETLRLHAPIHSIMRAVKTPMPVPGTKYVI
FG01000.1 	YQEQVKNLGADLPPLTYEDLAKLPLNQAIVKETLRLHAPIHSIMRAVKSPMPVPGTKYVI
AN8283.1  	YQEQLKVLGSDMH-MTYDDLQKLELHSKIIKETLRIHAPIHSIIRAVKSPMPVPGTSYVI
          	*:**:: **:*:  : *:** :* *:  ::*****:****:**:****  :*****.***

MG04432.1 	AKDTTVLAAPGYSAGDPNHFPEPELWEPHRWEADSRLAPRISMSNDNDEE-EKIDYGYGL
NCU02624.1	PTDHVLLAAPGVSATDESYFPQPDLWEPHRWEKDSPLAPTIVRNVPSEEDDEKIDYGYGL
FG01000.1 	PTSHTLLAAPGVSATDSAFFPNPDEWDPHRWEADSPNFPRMAS---KGEDEEKIDYGYGL
AN8283.1  	PTSHNVLSSPGVTARSDEFFPNPLKWDPHRWDSNP-----IAN---STEDEEKIDYGYGL
          	...  :*::** :* .  .**:*  *:****: :.     :     . *:.*********

MG04432.1 	VSKGTTSPYLPFGAGRHRCIGEHFANVQLQTIVAMIVREFKFRNVDGSGK--VVGTNYA~
NCU02624.1	VSKGANSPYLPFGAGRHRCIGEQFANVQLQTILAIIVRNFKFRNVDGSDK--VIGTDYA~
FG01000.1 	VSKGSASPYLPFGAGRHRCIGEHFANAQLQTIVAEVVREFKFRNVDGGHT--LIDTDYA~
AN8283.1  	VSKGTNSPYLPFGAGRHRCIGEQFAYVQLITVTAALVRLFKFDTVSESDKSSVPETDYS0
          	****: ****************:** .** *: * :** *** .*. . .:::  *:*: 

MG04432.1 	SLFSRPEEPAKIYWERR------
NCU02624.1	SLFSRPLEPAKIYWERREGCQL-
FG01000.1 	SLFSRPLEPANIHWERRQ-----
AN8283.1  	SLFSRPAGKCFVQYEKRNVTTKA
          	******   . : :*:*.    :
```
